# High-Resolution Analysis of Cytosine Methylation in Ancient DNA

**DOI:** 10.1371/journal.pone.0030226

**Published:** 2012-01-19

**Authors:** Bastien Llamas, Michelle L. Holland, Kefei Chen, Jennifer E. Cropley, Alan Cooper, Catherine M. Suter

**Affiliations:** 1 Australian Centre for Ancient DNA, University of Adelaide, Adelaide, South Australia, Australia; 2 Department of Ophthalmology, Flinders University, Bedford Park, South Australia, Australia; 3 Victor Chang Cardiac Research Institute, Darlinghurst, New South Wales, Australia; 4 Faculty of Medicine, University of New South Wales, Sydney, New South Wales, Australia; Institut de Biologia Evolutiva - Universitat Pompeu Fabra, Spain

## Abstract

Epigenetic changes to gene expression can result in heritable phenotypic characteristics that are not encoded in the DNA itself, but rather by biochemical modifications to the DNA or associated chromatin proteins. Interposed between genes and environment, these epigenetic modifications can be influenced by environmental factors to affect phenotype for multiple generations. This raises the possibility that epigenetic states provide a substrate for natural selection, with the potential to participate in the rapid adaptation of species to changes in environment. Any direct test of this hypothesis would require the ability to measure epigenetic states over evolutionary timescales. Here we describe the first single-base resolution of cytosine methylation patterns in an ancient mammalian genome, by bisulphite allelic sequencing of loci from late Pleistocene *Bison priscus* remains. Retrotransposons and the differentially methylated regions of imprinted loci displayed methylation patterns identical to those derived from fresh bovine tissue, indicating that methylation patterns are preserved in the ancient DNA. Our findings establish the biochemical stability of methylated cytosines over extensive time frames, and provide the first direct evidence that cytosine methylation patterns are retained in DNA from ancient specimens. The ability to resolve cytosine methylation in ancient DNA provides a powerful means to study the role of epigenetics in evolution.

## Introduction

Epigenetic changes to gene expression can produce variable and heritable phenotypes independent of either genetic or environmental factors. Most examples of heritable epigenetic variation have come from experimental models such as maize, *Arabidopsis* and mice [1], but epigenetic variation also occurs in natural systems [2]; in all cases a remarkable phenotypic divergence is created by variable epigenetic silencing of individual loci. Epigenetic states can be susceptible to environmental influence [3], and environmentally-induced epigenetic variants can also be inherited from one generation to the next [1]. This raises the possibility that environmentally-induced epigenetic changes in natural populations might produce new heritable phenotypes that can be acted upon by Darwinian selection [4], [5], providing a means of rapid adaptation to climate and environmental change without the requirement for DNA sequence alterations.

There is a growing interest in the potential evolutionary role of epigenetic variation and inheritance [5], [6], [7] but a direct test of this possibility would require the study of epigenetic marks over evolutionary timescales, by (for example) comparing the epigenotype of specimens that lived before with those that lived after some major environmental event. This would necessitate the study of epigenetic marks in ancient specimens, and would likely be limited to analysis of cytosine methylation: although the epigenetic state of a locus is defined by modifications to both DNA (i.e. methylation of cytosines) and its associated proteins, protein-based epigenetic modifications are not part of the DNA itself and are subject to rapid post-mortem decay.

The study of ancient DNA is itself not without challenges: it is usually found in limiting quantities, and is highly degraded and damaged. It is possible, however, to amplify single-copy nuclear sequences from well-preserved specimens. We supposed that the stability of cytosine methylation might allow it to persist in ancient specimens as long as the nuclear DNA remained intact. Indeed, the retention of cytosine methylation in ancient DNA has been implied by a recent study, but methylation was not assayed directly, and patterns were not resolved [8].

Here, we report the resolution of cytosine methylation patterns in a well-preserved ancient bovine specimen using bisulphite allelic sequencing. We find that the patterns of methylation at both imprinted genes and retrotransposons are remarkably similar to those observed in fresh bovine tissue. Our observations indicate that, providing nuclear DNA can be amplified, an accurate picture of cytosine methylation can easily be resolved from ancient templates.

## Results

### Species identification, DNA quality and dating of paleontological specimens

Fossilised remains of six Steppe bison (*Bison priscus*) were recovered from permafrost in the Yukon Territory, Canada (Table 1), and DNA extracted in a dedicated ancient DNA facility. A set of PCR amplicons of increasing size, targeting the mitochondrial control region (D-loop) of *B. priscus*, were generated from each sample; sequencing of the amplicons confirmed that the DNA in each sample was derived from *B. priscus*. In only one of the six samples (A3133) could the largest amplicon of 653bp be detected; this was also the only sample from which nuclear DNA could be amplified (Fig. S1). This sample was dated using accelerator mass spectrometry (Oxford Radiocarbon Accelerator Unit, Oxford, United Kingdom) to 26,360±220 uncalibrated radiocarbon years before present (Table 1).

**Table 1:** Description of the mummified and fossilised specimens and GenBank Accession of the mitochondrial control region sequences.

Sample ID	Tissue	Origin	Species	Sample age	Mito max PCR (bp)^e^	Nuclear locus PCR^f^	Accession^g^
A1176^a^	Skin	Takaka Hill, Nelson, New Zealand	*Bos taurus*	∼20^b^	278	Yes	N/A
A3133^a^	Astragalus	Irish gulch, Dawson, YT, Canada	*Bison priscus*	26,360±220^c^	653	Yes	HM443894
A5626	Metacarpal	Christie mine, Whitehorse, YT, Canada	*Bison priscus*	Late Pleistocene^d^	263	No	HM443895
A5627	Calcaneus	Irish Gulch, Dawson, YT, Canada	*Bison priscus*	Late Pleistocene^d^	278	No	HM443896
A5628	Calcaneus	Homesteak Gulch, Dawson, YT, Canada	*Bison priscus*	Late Pleistocene^d^	278	No	HM443897
A5629	Ulna	Gold Run, Whitman Creek, YT, Canada	*Bison priscus*	Late Pleistocene^d^	278	No	HM443898
A5630	Humerus	Gold Run, Whitman Creek, YT, Canada	*Bison priscus*	Late Pleistocene^d^	278	No	HM443899

a Specimens successfully amplified by bisulphite PCR;

b Estimated age in years;

c Uncalibrated radiocarbon dated years before present;

d Estimated, 10–60,000yrs BP;

e Longest PCR amplicon obtained targeting the mitochondrial control region;

f Success (Yes) or failure (No) of PCR targeting nuclear loci;

g GenBank accession of sequences of the mitochondrial control region.

We also employed two control samples: DNA extracted from a mummified modern cattle skin sample estimated to be around 20 years old, as an example of a modern but degraded template (spontaneous DNA decay occurring rapidly post-mortem [9]) and DNA extracted from fresh bovine tissue as an example of modern, undamaged template.

### Bisulphite allelic sequencing of ancient samples

We interrogated cytosine methylation patterns using bisulphite allelic sequencing [10]. Sodium bisulphite deaminates unmethylated but not methylated cytosines, and allelic methylation patterns can be resolved after PCR, cloning and sequencing. In mammals, methylation occurs almost exclusively at cytosine residues within CpG dinucleotides, and dense CpG methylation at gene promoters is characteristic of stable epigenetic silencing [11]. We targeted loci whose methylation patterns are well defined in mammals: multicopy retrotransposons (which are heavily methylated in somatic tissues) and single-copy differentially methylated regions (DMRs) associated with imprinted loci (which carry parent-of-origin specific methylation).

**Figure 1:**
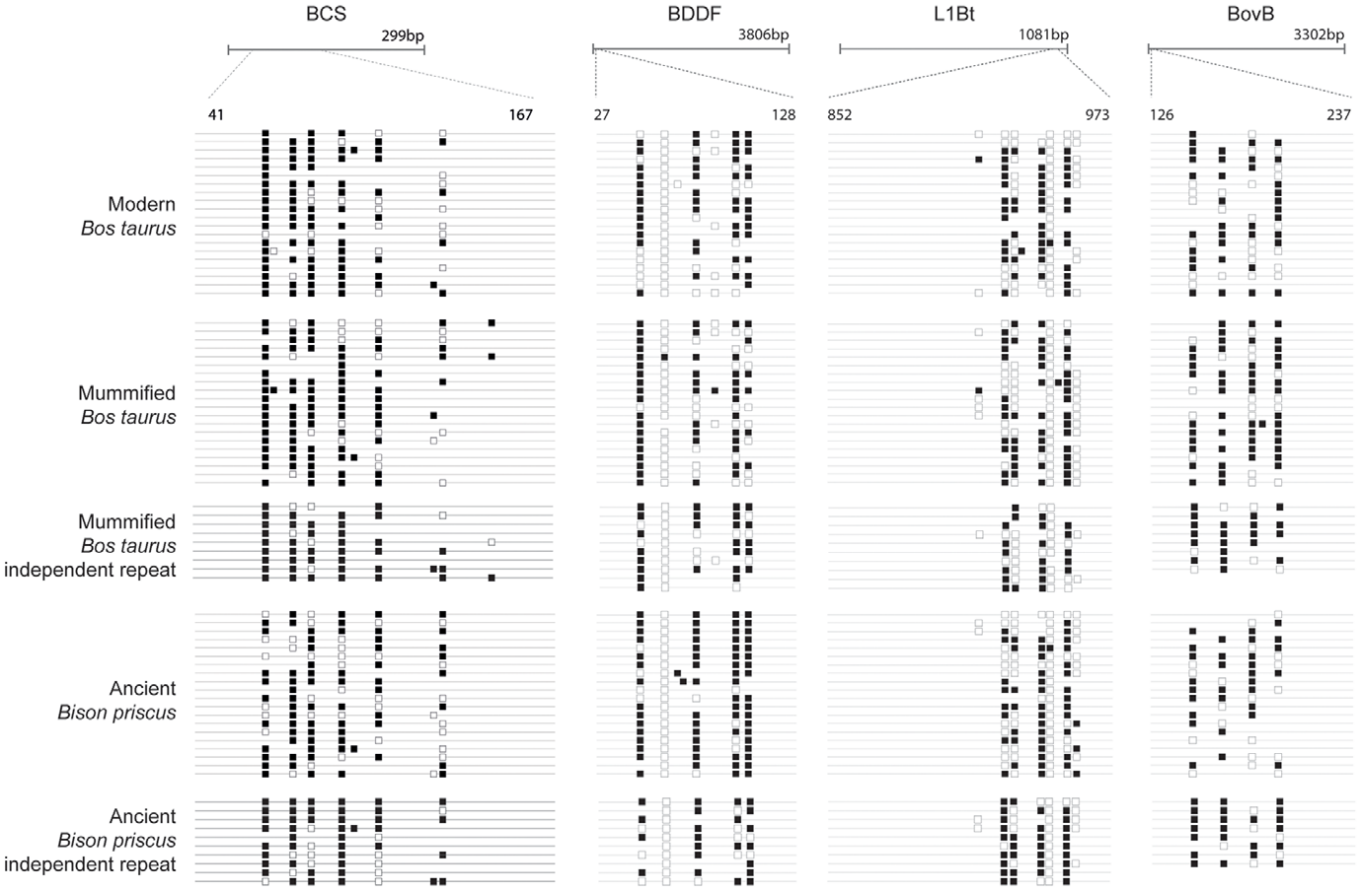
Methylation patterns of repetitive genomic elements in modern and ancient bovids. Maps of CpG methylation (white squares, unmethylated; black squares, methylated) composed of 20 individual clones (horizontal lines) were generated from *Bos taurus*, mummified *Bos taurus* and ancient *Bison priscus* targeting the multicopy retrotransposons as indicated above the maps. Differences in CpG placement within the retrotransposon maps are due to amplification of closely related but not identical elements from distinct locations within the genome. Retrotransposon consensus sequences were obtained from Repbase [21].

Bisulphite allelic sequencing maps for multicopy retrotransposon sequences are shown in Fig. 1. We readily amplified retrotransposon sequences from fresh and mummified DNA samples, but from only one of the six ancient DNA samples (sample A3133, that with the highest DNA integrity). DNA integrity appeared to be the defining factor in success of bisulphite PCR of the ancient sample; different bisulphite treatment protocols and extensive PCR optimisation failed to yield amplification from the five lower-quality samples. In ancient sample A3133 and the modern bovine samples, we detected the dense methylation patterns characteristic of retrotransposons. Replicate bisulphite PCR and sequencing, performed in spatially separated facilities on two independent A3133 DNA extractions, gave equivalent results (Fig. 1).

**Figure 2:**
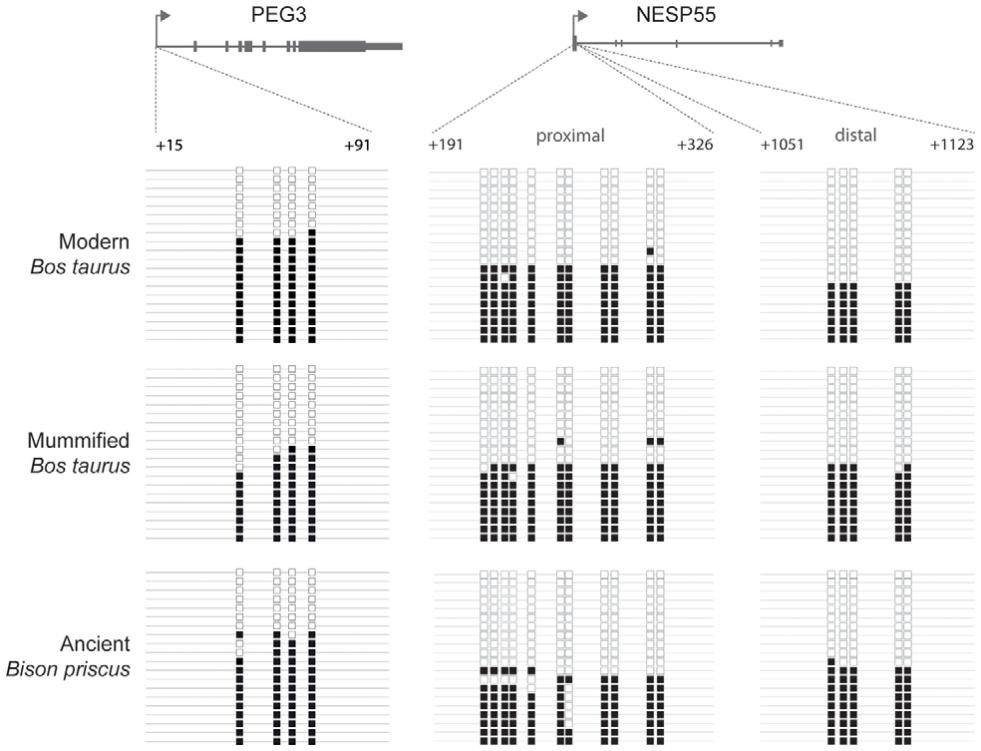
Methylation patterns of single copy imprinted genes in modern and ancient bovids. Maps of CpG methylation (white squares, unmethylated; black squares, methylated) composed of 20 individual clones (horizontal lines) were generated from *Bos taurus*, mummified *Bos taurus* and ancient *Bison priscus* targeting the single-copy imprinted genes *PEG3* (GenBank AY427787) and *NESP55* (GenBank U77614). Clone sequences are shown in Fig. S2, S3, S4.

Single-copy DMRs from the imprinted loci *PEG3* and *NESP55* were also readily amplifiable from fresh and mummified samples, and from ancient specimen A3133; bisulphite allelic sequencing maps are shown in Fig. 2 and sequence alignments in Figs. S2, S3, S4. In both the modern and ancient bovine DNA we found that approximately half the alleles were methylated and half unmethylated: this is consistent with the parent-of-origin specific methylation displayed by these loci in eutherian mammals [12], [13].

**Figure 3:**
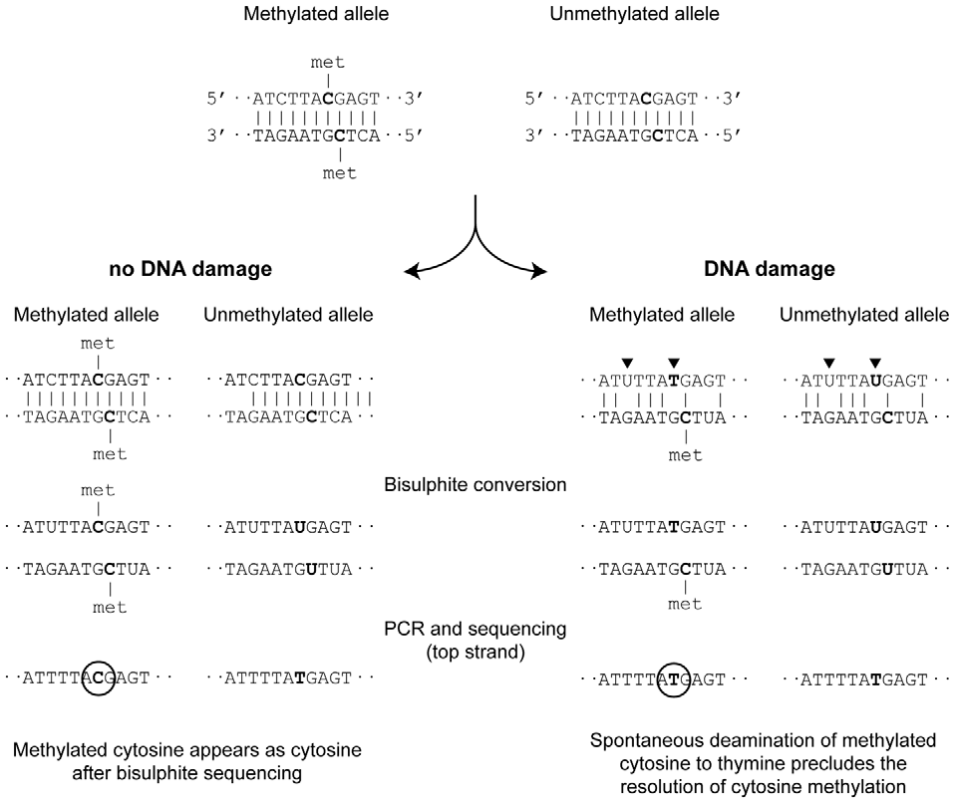
The potential effect of spontaneous post-mortem deamination of cytosine residues on the resolution of methylation patterns. In fresh samples (left), methylated cytosine is resistant to bisulphite conversion and can be resolved after PCR and sequencing. Post-mortem deamination (DNA damage) is highlighted by arrowheads in the ancient sample (right). A proportion of unmethylated cytosine in ancient templates is sometimes converted to uracil and appears as thymine following PCR amplification [14]; this would not affect the resolution of methylation. However, post-mortem deamination of methylated cytosine to thymine would prevent the detection of methylation via bisulphite sequencing (circles).

The potential impact of DNA damage on the accurate resolution of methylation patterns can be inferred from analysis of the single copy DMRs (the sequence divergence within families precludes this analysis at retrotransposons). The primary form of sequence-modifying template damage in ancient DNA is spontaneous deamination of cytosine [14], which would convert methylated cytosine to thymine, artifactually reducing the observed level of cytosine methylation upon bisulphite sequencing (Fig. 3). We found that DMR methylation patterns in the mummified and ancient samples mirror the fresh sample (Fig. 2 and Table S1), indicating that spontaneous deamination has not significantly altered the resolution of methylation patterns. There was one exception: the seventh CpG in the proximal *NESP55* DMR amplicon was converted in the majority of alleles (Fig. 2, Table S1). We thus examined the native (non- bisulphite treated) A3133 DNA for CG>TG transitions at this site, and at other CpGs across all three imprinted loci (Figs. S5–S7). The seventh CpG in the proximal *NESP55* DMR amplicon did not display a CG>TG transition in the native DNA (Fig. S6), excluding the possibility that spontaneous deamination is responsible for the pattern observed. It may be that the pattern is the result of biased representation of a certain allele in the bisulphite PCR, a situation that can be encountered with limiting template. Across the three imprinted loci, we observed a number of non-endogenous sequence artifacts such as A>G transitions [15] which had no impact on the resolution of methylation patterns; over the total of 310 CpG dinucleotides examined, we observed only six CG>TG transitions in the native ancient DNA (1.9%), and 15 other C>T transitions among the remaining 905 C residues (1.6%). Thus spontaneous deamination of CpG cytosines in ancient DNA appears uncommon and is unlikely to confound the resolution of methylation patterns.

## Discussion

Here we provide the first cytosine methylation patterns from the DNA of an ancient specimen. We found that the analysis of cytosine methylation was straightforward in this sample, which demonstrated high DNA integrity; unsurprisingly, we were unable to resolve cytosine methylation in samples from which nuclear DNA could not be amplified. Our results suggest that as long as ancient nuclear DNA remains amplifiable, cytosine methylation patterns can be assessed.

The methylation patterns we observed were consistent with those expected at retrotransposons and imprinted DMRs, indicating that cytosine methylation has been faithfully retained along with the DNA over evolutionary timescales. We found limited evidence for damage-related artifacts at CpG sites, indicating that, by and large, DNA damage-related artifacts do not interfere with the capacity to resolve methylation patterns in ancient DNA.

Epigenetic modifications have the potential to create phenotypic diversity in response to environmental cues, and unlike genetic changes, can be induced in multiple individuals in a population simultaneously. This would allow rapid adaptation to a changed environment, and in the face of intense selective pressure (as may be experienced during climatic change), pervasion of a phenotype throughout a population without any genetic change. The late Pleistocene was a time of dramatic climatic oscillations, and we have demonstrated that Steppe bison (as well as horse and other megafauna) from this period show marked morphological diversity but mitochondrial genetic homogeneity [16]. If epigenetics is responsible for these morphological changes, the challenge will be to identify the loci at which the epigenetic changes have occurred. Our data indicates that it is possible to obtain high-resolution picture of the methylation status of loci in well-preserved ancient specimens; whole-genome sequencing of ancient samples has also recently been demonstrated [17], [18], [19]. Together, these advances open the possibility for the correlation of DNA methylation patterns with genetics and morphology over evolutionary timescales using standard fossil remains and genome-wide approaches on next-generation sequencing platforms.

## Methods

### Ethics Statement

No specific permits were required for the described field studies.

### Paleontological samples and dating

A sample of skin was collected from a mummified *Bos taurus* specimen on Takaka Hill, NW Nelson, New Zealand. Ancient *Bison priscus* bones were recovered from permafrost in the Yukon Territory, Canada (Table 1). The ancient bison sample A3133 used for methylation analysis (below) was carbon-dated using accelerator mass spectrometry (Oxford Radiocarbon Accelerator Unit, Oxford, United Kingdom). This specimen dated to 26,360±220 uncalibrated radiocarbon years before present using a ^14^C half-life of 5,568 years.

### DNA extraction and assessment of degradation in ancient samples

DNA from fresh tissue was extracted from 500mg of bone marrow using standard proteolytic digestion and phenol/chloroform extraction at VCCRI (Victor Chang Cardiac Research Institute). Two separate DNA extractions from mummified and ancient samples were performed at ACAD (Australian Centre for Ancient DNA, University of Adelaide) in laboratories dedicated to low-copy number DNA research, using methods appropriate for ancient DNA research [20]. DNA from mummified tissue was extracted from 30mg of skin using Qiagen DNeasy Blood & Tissue kit (Qiagen) according to the manufacturer's protocol. DNA from six ancient bison was extracted from 500mg of powdered bone using proteolytic digestion and phenol/chloroform extraction followed by Amicon ultra-4 purification as previously described [16].

Genotyping of ancient samples was performed by PCR amplification of sequences from the mitochondrial control region (D-loop); all ancient mitochondrial sequences matched *Bison priscus* and the concatenated mitochondrial contigs have been deposited in GenBank (Table 1).

The extent of DNA degradation in mummified and ancient samples was assessed by PCR amplification of sequences of progressively increasing size from the mitochondrial control region (170, 263, 278 and 653bp). PCR was performed using 0.6 µM of each primer (see Table S2 for primer sequences), 250 µM dNTPs and 1.25U Platinum *Taq* Hi-Fidelity polymerase (Invitrogen), with the addition of 1mg/ml rabbit serum albumin (Sigma, fraction V) and 2mM MgSO_4_; thermal cycling parameters were 95°C for 2min, 50 cycles of 94°C for 20s, 55°C for 20s and 68°C for 30s, then 68°C for 10min. We were able to generate the 278bp amplicon from five of the six ancient samples, but the 653bp amplicon from only one well preserved ancient sample A3133 (Fig. S1). A3133 was also the only ancient sample from which we could amplify single-copy nuclear loci (Fig. S1). PCR conditions are as above and primer sequences are in Table S2.

### Methylation analysis by bisulphite allelic sequencing

Bisulphite treatment and PCR on DNA from fresh tissue was performed at VCCRI. Bisulphite treatment of two separately extracted series of DNA from mummified and ancient tissues was performed in the specialized low-copy number DNA research facilities at ACAD using the Epitect kit (Qiagen), and at VCCRI using the EZ DNA Methylation Kit™ (Zymo research). Bisulphite PCR was performed at ACAD; a replicate PCR was performed on the retrotransposon sequences at VCCRI. PCR was performed using the FastStart Taq system (Roche; see Table S3 for primer sequences); thermal cycling parameters were 95°C for 10min, 40 cycles of 94°C for 30s and 54°C for 30s, then 72°C for 5min. Cloning of products from all PCR reactions and sequencing of individual clones was performed at VCCRI.

### DNA damage analysis of the ancient bison A3133

For each of the two DNA extracts of the ancient bison A3133, duplicate PCR were performed at ACAD on native DNA using primers that were designed to amplify the same CpGs as were interrogated by bisulphite allelic sequencing (see Table S2 for primer sequences). PCR conditions were as described above for the single-copy nuclear loci. Cloning and sequencing of pooled duplicate PCR products were performed at VCCRI.

## Supporting Information

Figure S1 **Analysis of DNA integrity in mummified and ancient samples.** PCR amplification of (A) nuclear and (B) mitochondrial DNA from mummified *Bos Taurus* (A1176) and ancient bison (A3133, A5626-30) DNA. When sequenced, the mitochondrial products were all confirmed as *B. priscus.* Ext neg: DNA extraction negative control; PCR neg: no template control.
(TIF)

Figure S2 **Alignment of individual sequences from PEG3 bisulphite sequencing.** The reference sequence (top) is the *in silico* bisulphite converted PEG3 sequence (GenBank AY427787, nucleotides +44 to +67). Primer sequences are trimmed.
(TIF)

Figure S3 **Alignment of individual sequences from proximal NESP55 bisulphite sequencing.** The reference sequence (top) is the *in silico* bisulphite converted NESP55 sequence (GenBank U77614, nucleotides +217 to +302). The seventh CpG (asterisk) was abnormally converted in the majority of alleles in the ancient bison (See also Table S1). Primer sequences are trimmed.
(TIF)

Figure S4 **Alignment of individual sequences from distal NESP55 bisulphite sequencing.** The reference sequence (top) is the *in silico* bisulphite converted NESP55 sequence (GenBank U77614, nucleotides +1073 to +1099). Primer sequences are trimmed.
(TIF)

Figure S5 **Alignment of individual sequences from PEG3 sequencing.** The reference sequence (top) is the PEG3 sequence (GenBank AY427787, nucleotides +41 to +93). The black residues in the reference sequence are those interrogated by bisulphite allelic sequencing (Fig. S2), with arrowheads indicating cytosine residues potentially methylated. Primer sequences are trimmed.
(TIF)

Figure S6 **Alignment of individual sequences from proximal NESP55 sequencing.** The reference sequence (top) is the NESP55 sequence (GenBank U77614, nucleotides +218 to +317). The black residues in the reference sequence are those interrogated by bisulphite allelic sequencing (Fig. S3), with arrowheads indicating cytosine residues potentially methylated. Primer sequences are trimmed.
(TIF)

Figure S7 **Alignment of individual sequences from distal NESP55 sequencing.** The reference sequence (top) is the NESP55 sequence (GenBank U77614, nucleotides +1041 to +1120). The black residues in the reference sequence are those interrogated by bisulphite allelic sequencing (Fig. S4), with arrowheads indicating cytosine residues potentially methylated. Primer sequences are trimmed.
(TIF)

Table S1 **Statistical analysis of the ratio of methylated to unmethylated CpGs in single copy DMR loci.**
(DOCX)

Table S2 **Primer sequences for typing and assessment of DNA degradation in ancient samples.**
(DOCX)

Table S3 **Primer sequences for bisulphite PCR of retrotransposons and DMRs.**
(DOCX)
