# Supplementary figures and images for: High-Resolution Analysis of Cytosine Methylation in Ancient DNA
Source: PLoS One. 2012 Jan 19;7(1):e30226. doi: 10.1371/journal.pone.0030226 (PMC3261890; doi:10.1371/journal.pone.0030226)

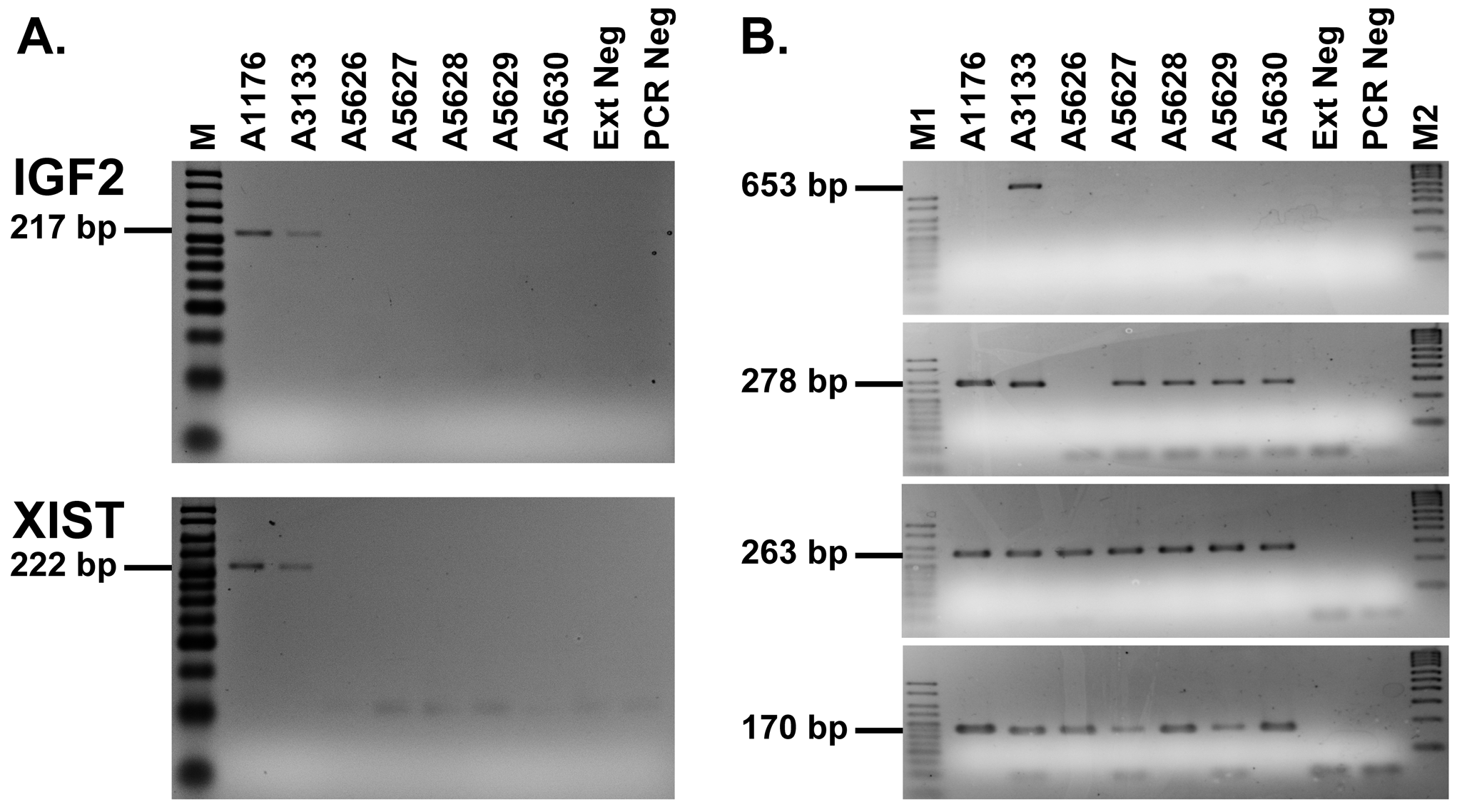

Supplement: Figure S1 — Analysis of DNA integrity in mummified and ancient samples. PCR amplification of (A) nuclear and (B) mitochondrial DNA from mummified Bos Taurus (A1176) and ancient bison (A3133, A5626-30) DNA. When sequenced, the mitochondrial products were all confirmed as B. priscus. Ext neg: DNA extraction negative control; PCR neg: no template control. (TIF) [file pone.0030226.s001.tif]

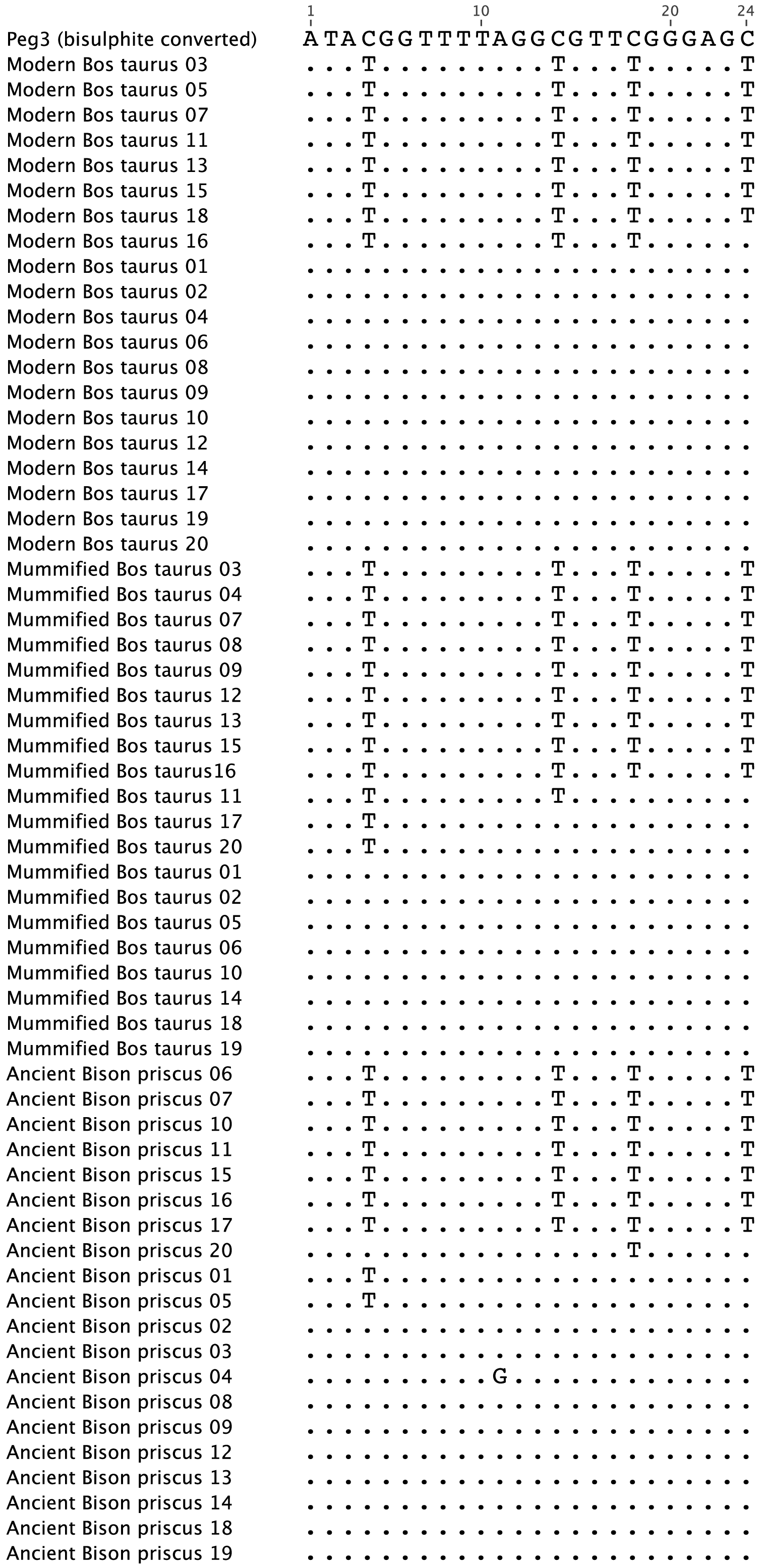

Supplement: Figure S2 — Alignment of individual sequences from PEG3 bisulphite sequencing. The reference sequence (top) is the in silico bisulphite converted PEG3 sequence (GenBank AY427787, nucleotides +44 to +67). Primer sequences are trimmed. (TIF) [file pone.0030226.s002.tif]

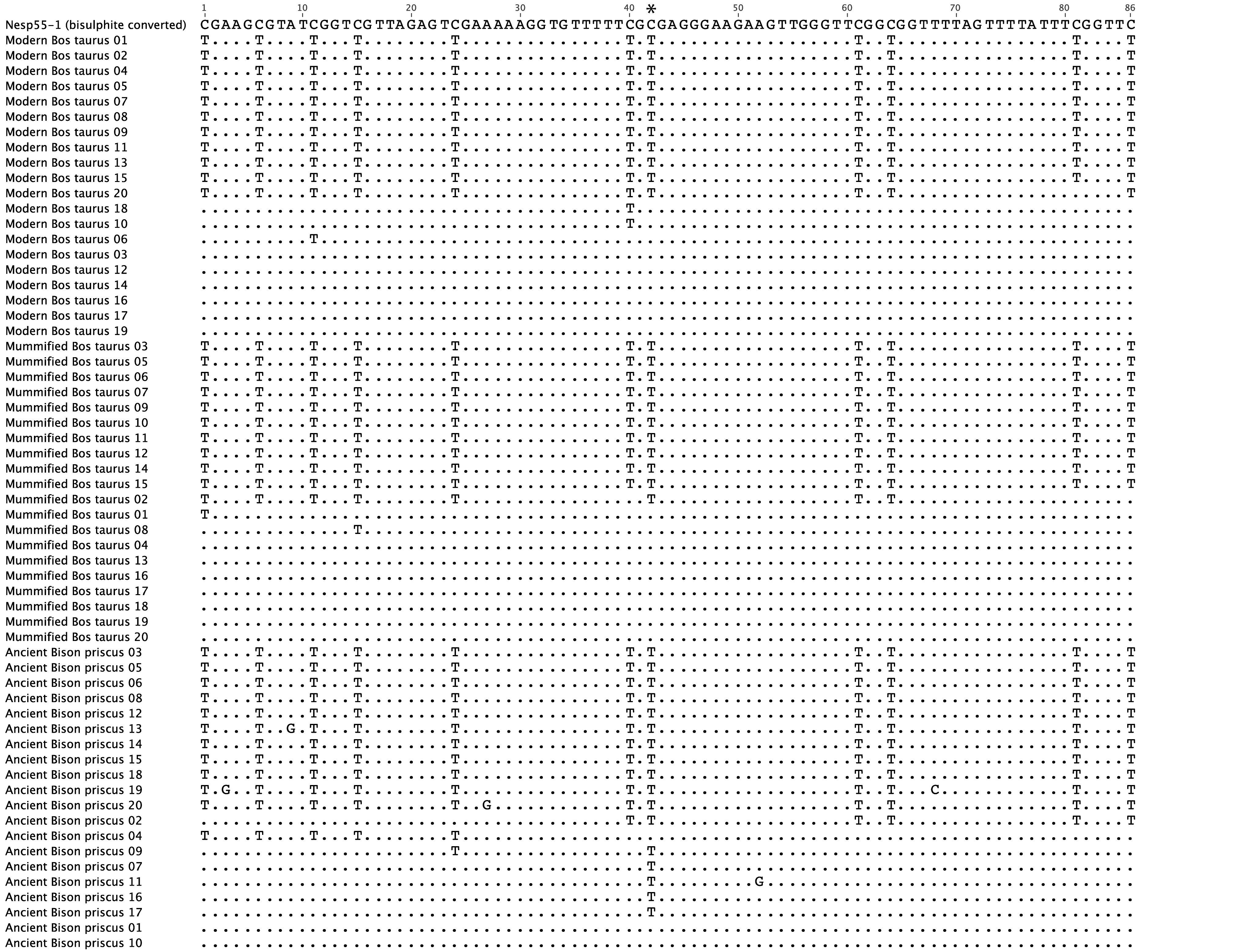

Supplement: Figure S3 — Alignment of individual sequences from proximal NESP55 bisulphite sequencing. The reference sequence (top) is the in silico bisulphite converted NESP55 sequence (GenBank U77614, nucleotides +217 to +302). The seventh CpG (asterisk) was abnormally converted in the majority of alleles in the ancient bison (See also Table S1). Primer sequences are trimmed. (TIF) [file pone.0030226.s003.tif]

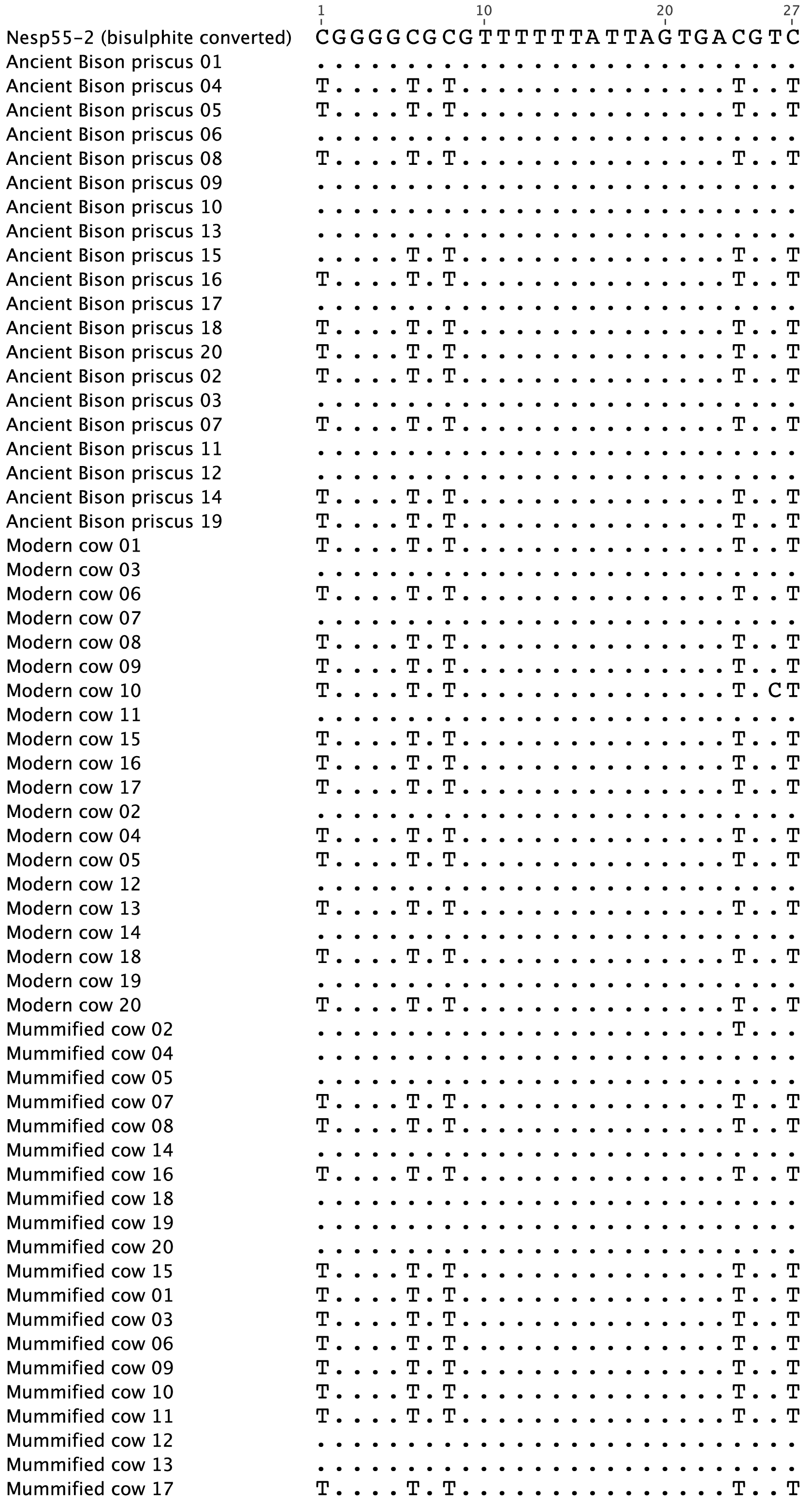

Supplement: Figure S4 — Alignment of individual sequences from distal NESP55 bisulphite sequencing. The reference sequence (top) is the in silico bisulphite converted NESP55 sequence (GenBank U77614, nucleotides +1073 to +1099). Primer sequences are trimmed. (TIF) [file pone.0030226.s004.tif]

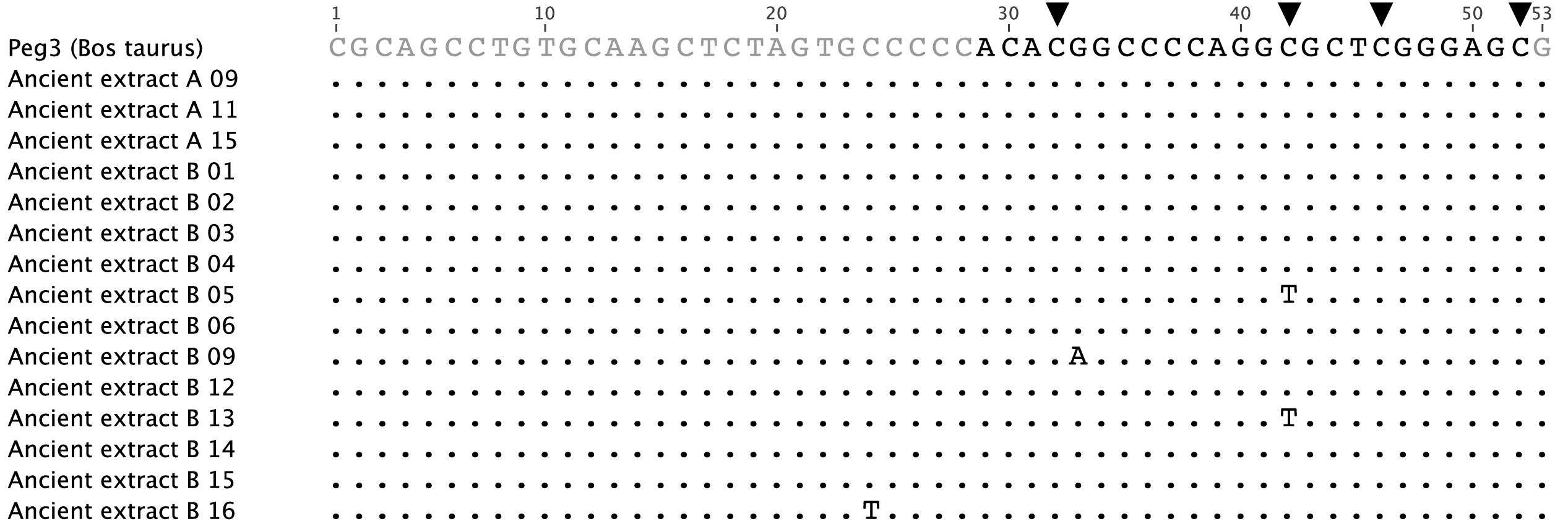

Supplement: Figure S5 — Alignment of individual sequences from PEG3 sequencing. The reference sequence (top) is the PEG3 sequence (GenBank AY427787, nucleotides +41 to +93). The black residues in the reference sequence are those interrogated by bisulphite allelic sequencing (Fig. S2), with arrowheads indicating cytosine residues potentially methylated. Primer sequences are trimmed. (TIF) [file pone.0030226.s005.tif]

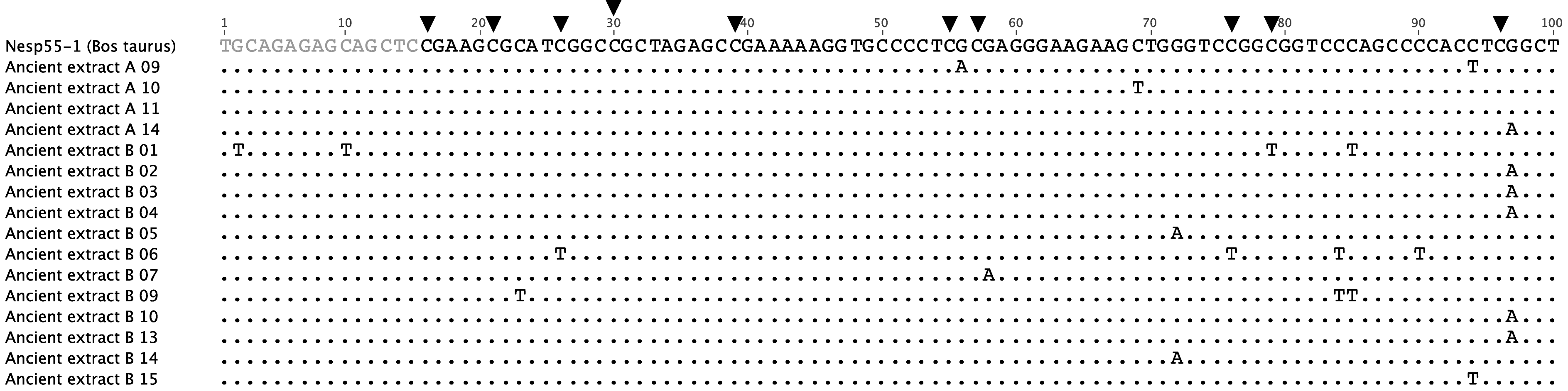

Supplement: Figure S6 — Alignment of individual sequences from proximal NESP55 sequencing. The reference sequence (top) is the NESP55 sequence (GenBank U77614, nucleotides +218 to +317). The black residues in the reference sequence are those interrogated by bisulphite allelic sequencing (Fig. S3), with arrowheads indicating cytosine residues potentially methylated. Primer sequences are trimmed. (TIF) [file pone.0030226.s006.tif]

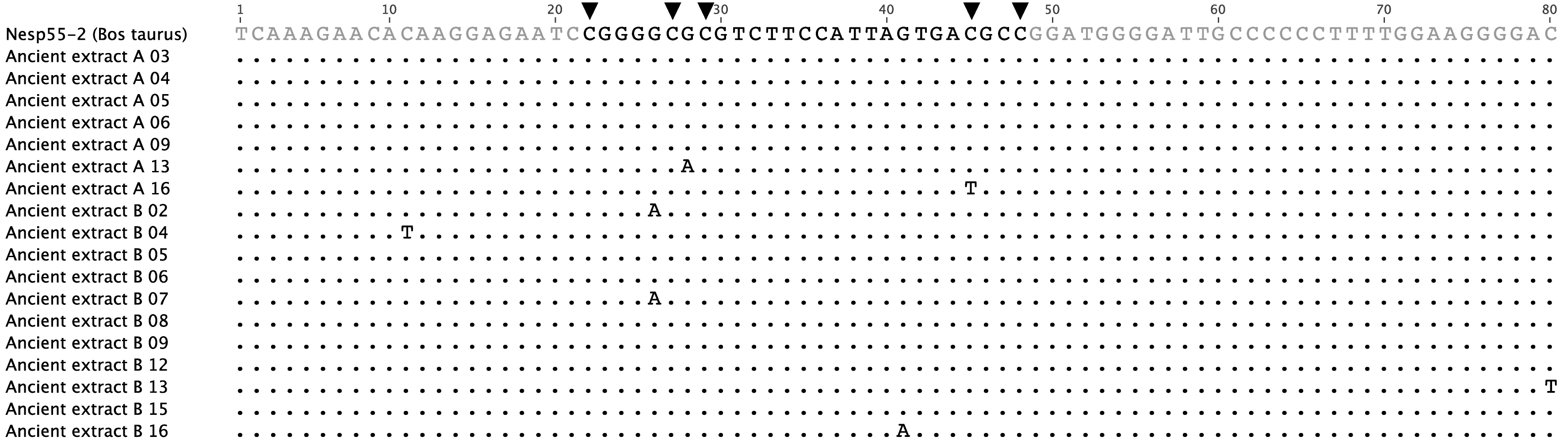

Supplement: Figure S7 — Alignment of individual sequences from distal NESP55 sequencing. The reference sequence (top) is the NESP55 sequence (GenBank U77614, nucleotides +1041 to +1120). The black residues in the reference sequence are those interrogated by bisulphite allelic sequencing (Fig. S4), with arrowheads indicating cytosine residues potentially methylated. Primer sequences are trimmed. (TIF) [file pone.0030226.s007.tif]
