# Supplementary material for: High-Resolution Analysis of Cytosine Methylation in Ancient DNA
Source: PLoS One. 2012 Jan 19;7(1):e30226. doi: 10.1371/journal.pone.0030226 (PMC3261890; doi:10.1371/journal.pone.0030226)
Supplement: Table S1 — Statistical analysis of the ratio of methylated to unmethylated CpGs in single copy DMR loci. (DOCX) [file pone.0030226.s008.docx]

**Table S1:** **Statistical analysis of the ratio of methylated to unmethylated CpGs in single copy DMR loci**

| **Locus** | **Methylation site** | **Modern *Bos taurus*** | | **Mummified *Bos taurus*** | | **Ancient *Bison priscus*** | |
| --- | --- | --- | --- | --- | --- | --- | --- |
|  |  | **Chi-square** | **P-value** | **Chi-square** | **P-value** | **Chi-square** | **P-value** |
| PEG3 | 1 | 0.8 | 0.3711 | 0.8 | 0.3711 | 0.2 | 0.6547 |
|  | 2 | 0.8 | 0.3711 | 0.0 | 1.0000 | 1.8 | 0.1797 |
|  | 3 | 0.8 | 0.3711 | 0.2 | 0.6547 | 0.8 | 0.3711 |
|  | 4 | 1.8 | 0.1797 | 0.2 | 0.6547 | 1.8 | 0.1797 |
| NESP55 (proximal) | 1 | 0.2 | 0.6547 | 0.8 | 0.3711 | 0.8 | 0.3711 |
|  | 2 | 0.2 | 0.6547 | 0.2 | 0.6547 | 0.8 | 0.3711 |
|  | 3 | 0.8 | 0.3711 | 0.2 | 0.6547 | 0.8 | 0.3711 |
|  | 4 | 0.2 | 0.6547 | 0.8 | 0.3711 | 0.8 | 0.3711 |
|  | 5 | 0.2 | 0.6547 | 0.2 | 0.6547 | 1.8 | 0.1797 |
|  | 6 | 0.2 | 0.6547 | 0.0 | 1.0000 | 0.8 | 0.3711 |
|  | 7 | 0.2 | 0.6547 | 0.2 | 0.6547 | 9.8 | 0.0017* |
|  | 8 | 0.2 | 0.6547 | 0.2 | 0.6547 | 0.8 | 0.3711 |
|  | 9 | 0.2 | 0.6547 | 0.2 | 0.6547 | 0.8 | 0.3711 |
|  | 10 | 0.0 | 1.0000 | 0.0 | 1.0000 | 0.8 | 0.3711 |
|  | 11 | 0.2 | 0.6547 | 0.0 | 1.0000 | 0.8 | 0.3711 |
| NESP55 (distal) | 1 | 1.8 | 0.1797 | 0.2 | 0.6547 | 0.0 | 1.0000 |
|  | 2 | 1.8 | 0.1797 | 0.2 | 0.6547 | 0.2 | 0.6547 |
|  | 3 | 1.8 | 0.1797 | 0.2 | 0.6547 | 0.2 | 0.6547 |
|  | 4 | 1.8 | 0.1797 | 0.8 | 0.3711 | 0.2 | 0.6547 |
|  | 5 | 1.8 | 0.1797 | 0.2 | 0.6547 | 0.2 | 0.6547 |

Chi-square: chi-square test of goodness of fit with 1 degree of freedom; P-value: two-tailed P-value, *P < 0.01. Methylation sites are numbered in the order they appear in Figure 2.
