# Supplementary material for: High-Resolution Analysis of Cytosine Methylation in Ancient DNA
Source: PLoS One. 2012 Jan 19;7(1):e30226. doi: 10.1371/journal.pone.0030226 (PMC3261890; doi:10.1371/journal.pone.0030226)
Supplement: Table S2 — Primer sequences for typing and assessment of DNA degradation in ancient samples. (DOCX) [file pone.0030226.s009.docx]

**Table S2: Primer sequences for typing and assessment of DNA degradation in ancient samples**

| **Amplicon** | **Primer** | **Primer Sequence (5' --- 3')** | **Length with primers** |
| --- | --- | --- | --- |
| CR_Frag1 | BovCR-16351F | CAACCCCCAAAGCTGAAG | 278 bp |
|  | BovCR-16652R | CTTGCTTATATGCATGGGGC |  |
| CR_Frag2 | BovCR-16633F^a^ | GCCCCATGCATATAAGCAAG | 170 bp |
|  | BovCR-16810R^a^ | GCCTAGCGGGTTGCTGGTTTCACGC |  |
| CR_Frag3 | BovCR-16765F^a^ | GAGCTTAAYTACCATGCCG | 263 bp |
|  | BovCR-80R^a^ | CAAGCATCCCCCAAAATAAA |  |
| CR_Frag4 | BovCR-16351F | CATCTGGTTCTTTCTTCAGGGCC | 653 bp |
|  | BovCR-80R^a^ | CAAGCATCCCCCAAAATAAA |  |
| IGF2 | Igf2-F | GATTAGAAGTGAGCCAAAGTGT | 217 bp |
|  | Igf2-R | GATGTTGTTCTGATCCCCTCAGC |  |
| XIST | Xist-F | TTTTGCTGCAGGGACAATATGGCTGAC | 222 bp |
|  | Xist-R | CCACCCTTTCTGATTGGGTGGGACAC |  |
| NESP55 (proximal) | Nesp55-1F | TCCGACCCACCCTCTGGCTC | 143 bp |
|  | Nesp55-1R | AGGTGCTTCCCTTTTTCCCCTCG |  |
| NESP55 (distal) | Nesp55-2F | CGGTGGGCAACCAACCTGGG | 120 bp |
|  | Nesp55-2R | CGCCGTGACCCATCCCCAGA |  |
| PEG3 | Peg3-F | GGCTGACAGCTCGACCACTG | 95 bp |
|  | Peg3-R | CATGTCCACCCTTGGCTGCTGG |  |

^a^ Primer sequences from Shapiro et al. [16]
