# Supplementary material for: High-Resolution Analysis of Cytosine Methylation in Ancient DNA
Source: PLoS One. 2012 Jan 19;7(1):e30226. doi: 10.1371/journal.pone.0030226 (PMC3261890; doi:10.1371/journal.pone.0030226)
Supplement: Table S3 — Primer sequences for bisulphite PCR of retrotransposons and DMRs. (DOCX) [file pone.0030226.s010.docx]

**Table S3: Primer sequences for bisulphite PCR of retrotransposons and DMRs**

| **Target** | **Primer** | **Primer Sequence (5' --- 3')^a^** | **Length** |
| --- | --- | --- | --- |
| BDDF | BDDF-F | ataactaaataacatcactcac | 102 bp |
|  | BDDF-R | CTTGCTTATATGCATGGGGC |  |
| BovB | BovB-F | tttaaggtaaggagtagtggttg | 112 bp |
|  | BovB-R | ctaataccctcttacaacacctac |  |
| L1Bt | L1Bt-F | tagattagttttatggattttgtgg | 145 bp |
|  | L1Bt-R | atacaccaatcccaaacatcc |  |
| BCS | BtBC-F | CATCTGGTTCTTTCTTCAGGGCC | 117 bp |
|  | BtBC-R | CAAGCATCCCCCAAAATAAA |  |
| NESP55 (proximal) | NESP55-1-F | TTTTTTGGTTTTGTAGAGAGTAGTTT | 137 bp |
|  | NESP55-1-R | AATAAATACTTCCCTTTTTCCCCTC |  |
| NESP55 (distal) | NESP55-2-F | TTGGGTTAAAGAATATAAGGAGAATT | 73 bp |
|  | NESP55-2-R | AAAAAAAACAATCCCCATCC |  |
| PEG3 | PEG3-F | GtAGttTGTGtAAGtTtTAGTGttttt | 76 bp |
|  | PEG3-R | AACATATCCACCCTTAACTACTAAC |  |

^a^ Based on Oct 2007 bovine genome assembly bosTau4
